# Mapping lymphatic filariasis morbidities in 24 endemic districts of Ethiopia through the health extension program

**DOI:** 10.1186/s41182-024-00657-6

**Published:** 2024-11-13

**Authors:** Haileleuel Bisrat, Fikre Hailekiros, Mebratu Mitiku, Asrat Mengiste, Merga Mekonnon, Fikre Seife, Birhanu Oljira, Haileyesus Terefe, Tamrat Bekele, Tsegahun Manyazewal

**Affiliations:** 1 Center for Innovative Drug Development and Therapeutic Trials for Africa (CDT-Africa), College of Health Sciences, Addis Ababa University, P.O. Box 9086, Addis Ababa, Ethiopia; 2 National Podoconiosis Action Network (NaPAN), Addis Ababa, Ethiopia; 3 Disease Prevention and Control Directorate, Federal Ministry of Health, Addis Ababa, Ethiopia; 4 Oromia Regional Health Bureau, Addis Ababa, Ethiopia; 5 Central Ethiopia Regional State Health Bureau, Werabe, Ethiopia; 6 Southwest Ethiopia Regional State Health Bureau, Tercha, Ethiopia

**Keywords:** Lymphatic filariasis, Morbidity management and disability prevention, Morbidity mapping, Lymphedema, Hydrocele, Ethiopia

## Abstract

**Background:**

The primary strategy for achieving the second goal of the Global Program to Eliminate Lymphatic Filariasis (GPELF) is morbidity management and disability prevention (MMDP), aimed at alleviating the suffering of affected populations. A significant challenge in many LF-endemic areas is the effective registration and identification of individuals with LF, which is crucial for planning and ensuring access to MMDP services. This study seeks to map the geographical distribution of LF-related morbidities across 24 endemic districts in Ethiopia.

**Methods:**

A community-based cross-sectional study was conducted to identify individuals affected by LF in 24 endemic districts using primary health care units (PHCUs). The study involved 946 trained health extension workers (HEWs) conducting house-to-house visits to identify and register cases of lymphedema and hydrocele, with support from 77 trained supervisors and 87 team leaders coordinating the morbidity mapping. Certified surgeons performed confirmatory evaluations through clinical assessments on a randomly selected sample of cases to validate HEW diagnoses, ensuring accurate identification of lymphedema and hydrocele. Statistical analysis of the data, including the severity of lymphedema and acute attacks, was conducted using STATA 17.

**Results:**

This study involved 300,000 households with nearly 1.2 million individuals, leading to the identification of 15,527 LF cases—14,946 (96.3%) with limb lymphedema and 581 (3.7%) with hydrocele. Among those with lymphedema, 8396 (54.1%) were women. Additionally, 13,731 (88.4%) patients resided in rural areas. Of the 14,591 cases whose acute attack information was recorded, 10,710 (73.4%) reported experiencing at least one acute attack related to their lymphedema in the past 6 months, with a notable percentage of males (74.5%; *n* = 4981/6686). Among the 12,680 recorded cases of leg lymphedema, the percentage of acute attacks increased with severity: 64% (*n* = 5618) mild cases, 68% (*n* = 5169) moderate cases and 70% (*n* = 1893) severe cases.

**Conclusion:**

This study successfully mapped the geographical distribution of LF morbidities across 24 LF-endemic districts in Ethiopia, identifying a substantial number of lymphedema and hydrocele cases, particularly in rural areas where healthcare access is limited. The findings underscore the potential of Ethiopia’s health extension program to identify affected individuals and ensure they receive necessary care. The findings inform targeted interventions and access to MMDP services, contributing to Ethiopia’s goal of eliminating LF by 2027.

## Introduction

Lymphatic filariasis (LF), caused by parasitic nematodes such as *Wuchereria bancrofti, Brugia malayi, and Brugia timori*, is a debilitating neglected tropical disease (NTD) that affects around 70 million people globally [1–3]. The disease predominantly impacts countries in the Global South, where factors like environmental conditions, poverty, and inadequate healthcare systems facilitate LF transmission [4]. Over 80% of global LF cases are concentrated in sub-Saharan Africa, South Asia, and Southeast Asia [4]. LF is transmitted by various mosquito species, including Culex, Anopheles, and Aedes, which thrive in tropical and subtropical climates [5]. The parasitic worms reside in the human lymphatic system, leading to progressive damage and chronic conditions such as lymphedema (tissue swelling), elephantiasis (severe swelling and skin thickening), and hydrocele (scrotal swelling) [6]. Without effective intervention, these conditions can result in long-term disability, social stigma, and economic hardship, particularly in low-resource settings with limited healthcare access [6].

In the broader Global South, efforts to eliminate LF are hindered by socioeconomic challenges, including poverty, inadequate healthcare infrastructure, and high population density in endemic areas [7]. Despite global initiatives, many low- and middle-income countries (LMICs) face significant obstacles in implementing mass drug administration (MDA) and morbidity management and disability prevention (MMDP) programs [8]. Countries like India, Tanzania, and Bangladesh, despite their large-scale national programs, continue to struggle with achieving sufficient MDA coverage, ensuring adherence, and providing follow-up care for those affected [9, 10].

Ethiopia is one of the Africa global countries severely affected by LF. Ethiopia's efforts to combat lymphatic filariasis began slowly, starting with MDA in just five districts of the Gambella region, covering only 7% of the area in 2009 [11]. The MDA program aims to interrupt LF transmission by delivering antifilarial medications annually, specifically ivermectin and albendazole, to at-risk populations in endemic areas through a community-based approach involving health extension workers (HEWs) and community volunteers [12]. The country has been conducting large-scale nationwide MDA for various NTDs since 2007. Noncompliance with the MDA program has been linked to specific demographic, individual, programmatic, and drug delivery factors [13]. Additionally, the MDA program includes MMDP for individuals already affected by LF, addressing the needs of those suffering from lymphedema and hydrocele [14]. Despite challenges such as geographic accessibility and logistical issues, MDA coverage has steadily increased, resulting in a significant reduction in LF prevalence [13, 15]. Integrating a community-based holistic care package that addresses physical and psychosocial needs into the Ethiopian health system has shown the promise to reduce morbidity among individuals living with LF [16].

Mapping the distribution of LF-related morbidities is essential for efficient resource allocation and ensuring access to MMDP services [15]. Like many LMICs, Ethiopia faces barriers to eliminating LF due to limited healthcare infrastructure and the dispersed nature of rural populations [16]. However, by leveraging its health extension program, Ethiopia is adapting successful models from other LMICs while addressing its unique geographical and demographic challenges [17]. The Ministry of Health (MoH) of Ethiopia, in collaboration with global partners like the END FUND, is focusing on mapping endemic districts and identifying patients in need of MMDP services.

Therefore, this study aimed to map the geographical distribution of LF morbidities across 24 endemic districts in Ethiopia to improve identification of affected individuals and ensure they receive the necessary care.

## Methods

### Study design

A community-based cross-sectional study was conducted through the HEW network to identify cases. Trained HEWs performed house-to-house visits in each targeted district. Since its launch in 2006, the Ethiopian Health Extension Program has established a network of over 70,000 community-based HEWs, supported by a supervisory framework. These healthcare workers were strategically positioned to carry out comprehensive screenings and register cases of lymphedema and hydrocele during their visits in designated areas.

### Study area

**Fig. 1:**
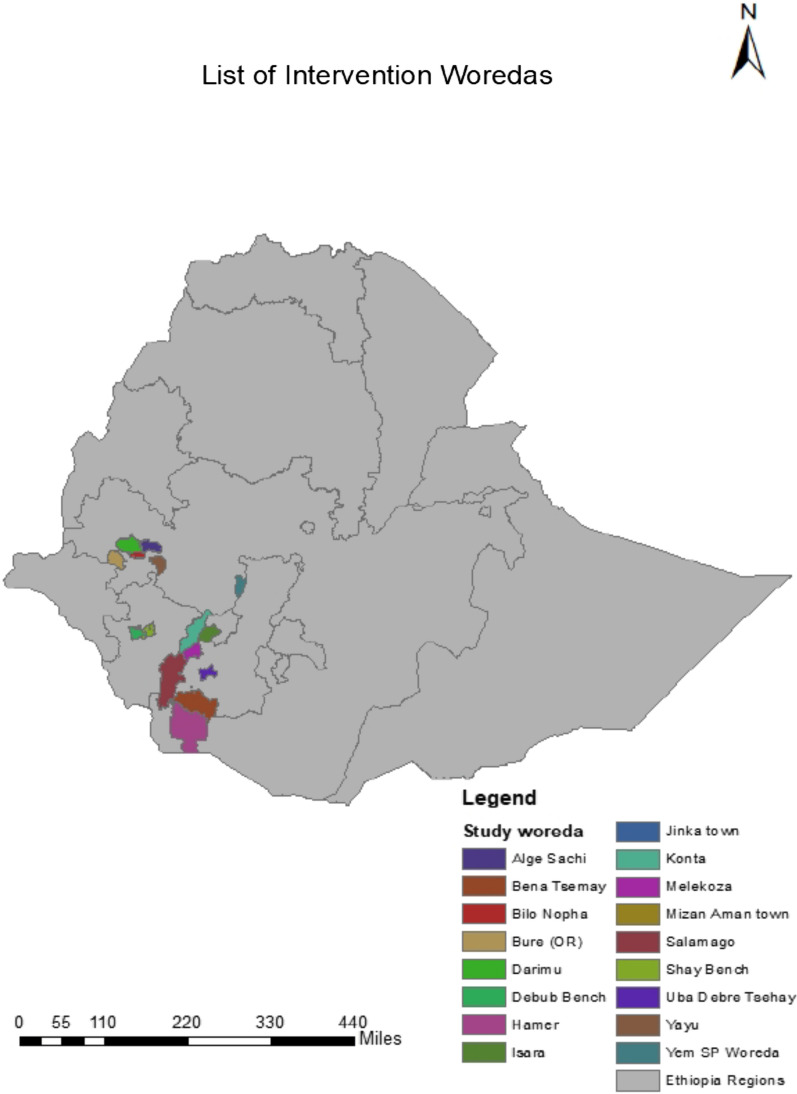
Study area map of the 24 districts. *Note* Konta and Yem zones were newly split into different districts so the new district cannot see in the map. which is In Konta zone: Ameya Town, Ameya Zuria, Elahanchano, Chida and Konta Koisha district in Yem zone: Saja town, Saja zuria, Fofa and Tpba district

The study took place from August 28, 2023, to October 26, 2023, across four regions of Ethiopia: Southern Ethiopia, southwestern Ethiopia, Central Ethiopia, and the Oromia regions (Fig. 1). A total of 24 districts were selected, including Jinka, Benatsemay Selamago, Hamer, Uba Debretsehay, and Melekoza in southern Ethiopia; Esera, Ameya town, Ameya zoria, Elahanchano, Chida, Konta Koisha, Mizan Aman town, and South Bench in southwestern Ethiopia; Saja town, Saja Zuriya, Fofa, and Toba in central Ethiopia; and Alge Sach, Bilo Nopa, Bure, Darimu, and Yayo in the Oromia region. Every household in these endemic areas was visited to compile a comprehensive list of all cases. The districts were chosen in collaboration with the Ethiopian MOH and Regional Health Bureaus due to their endemic status for LF and because they were among the few remaining areas where morbidity mapping had not yet been completed.

### Participants

In each district, at least two HEWs per health post participated in data collection. HEWs are government-employed community health workers operating within Ethiopia’s primary health care units (PHCUs), which form the foundation of the healthcare system and provide basic health services to rural and underserved communities. PHCUs typically consist of a health center and satellite health posts, with HEWs managing health service delivery at the community level.

The data collection involved door-to-door interviews conducted over ten consecutive days in each district. To minimize disruption to the HEWs' routine responsibilities, their participation was coordinated with local health officials. Since HEWs are government employees, their involvement was integrated into their regular duties. Prior to the data collection, HEWs received additional training on identifying and registering cases of lymphedema and hydrocele, ensuring they could efficiently carry out their tasks without neglecting their routine health post duties.

Supervisors and team leaders provided logistical support and oversight, ensuring data collection was completed within the 10-day timeframe while maintaining the quality of regular health services at health posts. Collaboration between PHCUs and district health offices was crucial in enabling HEWs to effectively balance their study-related duties with their routine responsibilities.

### Ethics statement

Ethical approval for this study was granted by the ethical review committees in each region. Informed consent was obtained from all household heads and patients involved in the study. Participants who consented were registered and asked to sign or provide a fingerprint on the consent form. Individual written informed consent was collected from each participant aged 18 and older. For participants under 18, consent was obtained from their parents or guardians, while the young participants themselves provided informed assent.

### Statistical analysis

All data were entered into Microsoft Excel Version 12.3.6 (Microsoft Corp., Redmond, VA, USA), and analysis was performed using STATA 17 (StataCorp). Each participant was assigned a unique ID, allowing for the merging of datasets before analysis. Prevalence estimates (per 10,000 population) were calculated using the 2015 population figures derived from the 2007 census [18] and adjusted for annual growth rates [19]. Statistical analyses were conducted to compare regions and variables, including disease condition, severity of lymphedema, acute attacks, sex, and age. Confirmatory assessments were carried out in each zone to validate the results.

## Results

### Background characteristics

This study involved 300,000 households and nearly 1.2 million individuals, leading to the identification of 15,527 cases—14,946 (96.3%) of lymphedema and 581 (3.7%) of hydrocele—through door-to-door interviews conducted between August 28 and October 26, 2023. Among the identified cases, 8396 (54.1%) were female, with 51% under 40 years of age and 49% aged 40 or older. Additionally, 10,137 participants (65.3%) were illiterate, 11,001 (70.8%) were married, and 9402 (61%) worked as farmers. Other occupations included 3142 (20%) housewives, 662 (4%) day laborers, 655 (4%) in various roles, and the remaining 1666 (11%) were categorized as others (Table 1).

**Table 1:** Background characteristics of the study participants (*n* = 15,527)

Variable	Number	%
*Sex*		
Male	7131	45.9
Female	8396	54.1
*Residence*		
Rural	13,731	88.4
Urban	1796	11.6
*Marital status*		
Single	2253	14.5
Married	11,001	70.8
Separated	543	3.5
Divorced	430	2.8
Widowed	1300	8.4
*Educational status*		
Do not read and write	10,137	65.3
Not attended formal education but can read and write	889	5.7
Grade 1–4	2089	13.5
Grade 5–8	1251	8.1
Other	1161	7.4

Table 2 presents the number of cases reported per clinical condition for each region, zone and district. For the total of 15,527 cases reported, the cases are distributed as follows: Ari zone 41 cases (41.5% male; mean age 46.9 years), South Omo Zone 285 cases (69.2% male; mean age 41.0 years), Bench Seko Zone 2564 cases (40.5% male; mean age 40.6 years), Dawro Zone 436 cases (51.6% male; mean age 47.1 years), Gofa Zone 1384 cases (57.3% male; mean age 43.4 years), Konta Zone 1130 cases (56.8% male; mean age 46.4 years), Yem Zone 984 cases (52.6% male; mean age 45.8 years) and Illu Aba Bora Zone 8703 cases (43.6% male; mean age 45.9 years), which had the highest number of reported patients. In terms of clinical conditions, the total number of cases reported was 15,527 (96.3%) with leg lymphedema and 581 (3.7%) with hydrocele. No individual was reported to have both leg lymphedema and hydrocele.

**Table 2:** Reported number and prevalence (per 10,000 of the total population) of clinical cases

Region	Zone	#	District	Total population	Lymphedema		Hydrocele		Both conditions	
					*N*	Prevalence	*N*	Prevalence	*N*	Prevalence
	Ari	1	Jinka	34,723	38	10.9	3	0.9	41	11.8
Ari overall total				34,723	38	10.9	3	0.9	41	11.8
South Ethiopia Region	South Omo	2	Selamago	40,338	114	28.3	25	6.2	139	34.5
	South Omo	3	Benatsemay	76,647	52	6.8	23	3.0	75	9.8
	South Omo	4	Hammer	86,394	37	4.3	34	3.9	71	8.2
	South Omo overall total						82		285	
	Gofa	5	Uba Debretsehay	87,761	501	57.1	25	2.8	526	59.9
	Gofa	6	Melekoza	120,907	822	68.0	36	3.0	858	71.0
	Gofa overall total						61		1384	
South Ethiopia Region overall total				412,047	1526	35	143	3.2	1669	37.4
South West Ethiopia Region	Dawro	7	Esera	94,329	425	45.1	11	3.2	436	46.2
	Dawro overall total						11		436	
	Konta	8	Ameya Town	20,408	232	113.7	17	1.2	249	122.0
	Konta	9	Ameya Zuria	40,430	274	67.8	20	8.3	294	72.7
	Konta	10	Elahanchano	27,516	150	54.5	6	4.9	156	56.7
	Konta	11	Chida	14,920	158	105.9	0	2.2	158	105.9
	Konta	12	Konta Koisha	30,896	258	83.5	15	0.0	273	88.4
	Konta overall total						58		1130	
	Bench Sheko	13	Mizan Aman Town	85,680	336	39.2	5	4.9	341	39.8
	Bench Sheko	14	South Bench	134,536	990	73.6	18	0.6	1008	74.9
	Bench Sheko	15	Shey Bench	149,068	1195	80.2	20	1.3	1215	81.5
	Bench Sheko overall total						43		2564	
South West Ethiopia Region overall total				597,783	4018	67.2	112	1.9	4130	69.1
Central Ethiopia Region	Yem	16	Saja town	13,502	203	150.3	3	2.2	206	152.6
	Yem	17	Saja Zuria	28,549	263	92.1	9	3.2	272	95.3
	Yem	18	Fofa	57,015	340	59.6	16	2.8	356	62.4
	Yem	19	Toba	27,122	147	54.2	3	1.1	150	55.3
Central Ethiopia Region overall total				126,188	953	75.5	31	2.5	984	78.0
Oromia	Illu Aba Bora	20	Alge Sach	119,457	1537	128.7	53	4.4	1590	133.1
	Illu Aba Bora	21	Bilo Nopa	44,610	784	175.7	17	3.8	801	179.6
	Illu Aba Bora	22	Bure	79,522	1975	248.4	55	6.9	2030	255.3
	Illu Aba Bora	23	Darimu	222,189	2620	117.9	134	6.0	2754	123.9
	Illu Aba Bora	24	Yayo	83,439	1495	179.2	33	4.0	1528	183.1
Oromia overall total				549,217	8411	153.1	292	5.3	8703	158.5
Overall total				1,719,958	14,946	86.9	581	3.4	15,527	90.3

**Fig. 2:**
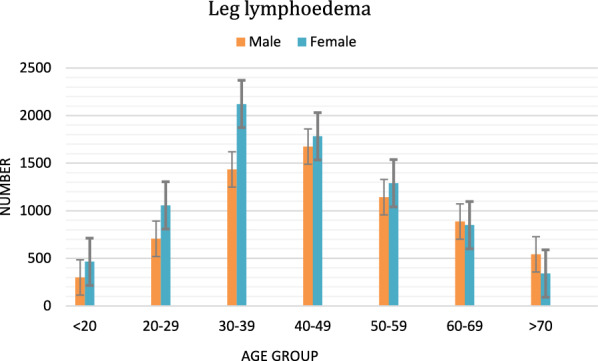
Leg lymphedema and hydrocele by age and sex

Figure 2 illustrates the distribution of leg lymphedema and hydrocele by age and sex.

### Acute attacks

Of the 15,527 identified cases, 936 lacked reliable information regarding acute attacks in the last 6 months and were excluded from this analysis (Table 3). Among the remaining 14,591 patients, 10,710 (73.4%) reported experiencing at least one acute attack in the past 6 months related to swelling or lymphedema. A higher percentage of males reported having an acute attack during this period (74.5%; *n* = 4981 out of 6686) compared to females (72.5%; *n* = 5729 out of 7905).

**Table 3:** Reported acute attacks for all conditions by different age groups and by sex

Overall			Differences by sex			
Age group	Total cases	No. positive (%)	Sex	Subtotal (*n*)	Total positive	Positive %
< 20	763	544 (71.3)	M	299	212	70.9
			F	464	332	71.6
20–29	1763	1318 (74.8)	M	706	548	77.6
			F	1057	770	72.8
30–39	3557	2658 (74.7)	M	1435	1112	77.5
			F	2122	1546	72.9
40–49	3457	2558 (74.0)	M	1674	1264	75.5
			F	1783	1294	72.6
50–59	2432	1758 (72.3)	M	1143	826	72.3
			F	1289	932	72.3
60–69	1736	1251 (72.1)	M	887	639	72.0
			F	849	612	72.1
> 70	883	623 (70.6)	M	542	380	70.1
			F	341	243	71.3
Total	14,591	10,710 (73.4)	M	6686	4981	74.5
			F	7905	5729	72.5

NB: 936 participants did not record information on acute attacks and were therefore excluded from the analysis

Positive refers to patients who reported experiencing at least one acute attack in the last 6 months

M, male; F, female

### Leg lymphedema

Information on the severity of leg lymphedema was recorded for 12,680 patients. The data showed that the percentage of reported acute attacks increased with the severity of the condition: mild cases had a rate of 64% (*n* = 5618), moderate cases had 68% (*n* = 5169), and severe cases reported 70% (*n* = 1893) (Table 4).

**Table 4:** Severity of reported leg lymphedema and acute attacks in reported cases

Severity	Overall		Difference by sex			
	Total case	No. positive	Sex	Subtotal	Total positive	Positive
Mild	5618	3596 (64%)	M	2502	1524	60.9
			F	3116	2072	66.5
Moderate	5169	3514 (68%)	M	2287	1459	63.8
			F	2882	1955	67.8
Severe	1893	1325 (70%)	M	866	627	72.4
			F	1027	698	68.0
Total	12,680		M	5655	3610	63.8
			F	7025	4725	67.3

M, male; F, female

The ordered logistic regression for the severity of acute attacks with other variables demonstrate that residence, occupation and age have significant association with severity of acute attacks (Table 5).

**Table 5:** Ordered logistic regression for the severity of acute attacks with other variables

Severity	*P* >|*z*|	(95% conf. interval)
Sex	0.941	(− 0.0716263 0.0664009)
Residence	0.048	(0.0010738 0.2048152)*
Occupation	0.000	(− 0.0004154 − 0.0001227)***
Education_level	0.325	(− 0.00011 0.0003318)
Marital status	0.826	(− 0.0388078 0.030986)
Age	0.000	(0.0303703 0.0759607)***

Abbreviations: ordered logistic regression; 95% CI; 95% confidence interval

***p value < 0.001; **0.001 ≤ *p* value <0.01; *0.01 ≤−*p* value <0.05

A significant majority of patients, 64%, reported having swollen legs for 1–10 years, indicating a high prevalence of more recent cases. In contrast, among those who have had swollen legs for 10–20 years, the prevalence drops significantly to 25%, suggesting a decrease in the proportion of cases over this longer duration (Table 6).

**Table 6:** Years of leg swelling experience

S. no.	Number of years patient live with swollen leg	Proportion of people with lymphedema (%)
1	1–10	64
2	10–20	25
3	> 20	11

### Confirmatory test

Following the initial assessment, 96 cases were subjected to confirmatory evaluation. Ten cases of lymphedema and two cases of hydrocele were collected from randomly selected districts. The results showed that 80% of the hydrocele cases matched the assessments made by the HEWs, indicating a strong level of agreement. Similarly, 95% of the lymphedema cases were consistent with the HEW evaluations.

## Discussion

This study represents a comprehensive community-wide clinical case survey of LF in 24 endemic areas in Ethiopia. Recent survey findings emphasize a significantly greater prevalence of lymphedema cases than hydrocele cases, with leg lymphedema cases outnumbering hydrocele cases by more than 24 times. In a separate study, this disparity was even more pronounced, with 33 times as many reported leg lymphedema cases than hydrocele cases [20]. Contrasting data from studies conducted in LF-endemic regions such as Tanzania and Malawi revealed a different trend, with the number of hydrocele cases nearly doubling the number of lymphedema cases identified [21, 22]. This disparity is likely influenced by the presence of podoconiosis in Ethiopia.

The predominant observation from this study was the bilateral manifestation of the majority of lymphedema cases, a characteristic more commonly associated with nonfilarial lymphedema [23]. While these findings suggest that a significant portion of identified lymphedema cases could be attributed to podoconiosis rather than filariasis, the study did not differentiate between the underlying causes of lymphedema. These results align with earlier research by Deribe et al. [24], underscoring the substantial prevalence of podoconiosis in the Southern Nations, Nationalities, and Peoples' Region and Amhara region of Ethiopia, particularly in the central highland areas where environmental conditions favor the occurrence of podoconiosis. In LF and podoconiosis co-endemic regions, diagnostic tests such as circulating filarial antigen testing, filarial antibody examination, and parasitological examination have been employed to rule out LF diagnosis [25]. However, in the context of this study, which focused on establishing MMDP interventions for assessing the burden of lymphedema, a comprehensive understanding of the etiology was not deemed necessary. Both filarial and nonfilarial lymphedema patients require similar MMDP interventions, emphasizing the importance of addressing the burden of lymphedema regardless of its underlying cause. Irrespective of the underlying causes, the significant prevalence of leg lymphedema cases underscores the critical necessity of providing essential care to individuals affected by these incapacitating conditions, particularly in regions with a high incidence or concentration of cases where patients can be more easily located and where care distribution can be facilitated. The implementation of a cost-effective lymphedema management program centered on limb hygiene and topical treatments for infections has demonstrated efficacy in reducing the frequency of distressing acute episodes and enhancing the economic productivity of patients [26]. A comprehensive MMDP initiative is poised to benefit the majority of lymphedema cases in these areas, given that most cases are categorized as mild and are likely to respond positively to such interventions [27]. This integrated MMDP program should seamlessly integrate into the existing healthcare infrastructure to ensure longevity and contribute to achieving universal health coverage. Furthermore, early identification of mild lymphedema cases, which might be underreported by HEWs, should be emphasized to impede the progression to more severe stages of lymphedema.

In previous research endeavors, a verification process involving clinical assessment by a healthcare provider was employed to validate the accuracy of reported cases of lymphedema and hydrocele identified during patient screening [28, 29]. In the current study, following the initial evaluation, a confirmatory assessment was carried out in each zone to corroborate the findings. The results revealed a substantial agreement level, with 80% of hydrocele cases corresponding with the assessments conducted by HEWs. Likewise, 95% of the lymphedema cases were in concordance with the assessments made by HEWs, indicating a high level of consistency in the reported cases.

The study results indicated that individuals with more severe disease were at a greater risk of experiencing acute attacks in the past 6 months. This observation aligns with findings from a prior study conducted in the same country, where individuals with more severe disease presentations were also found to have a greater likelihood of experiencing acute attacks [20].

The low number of hydrocele cases identified in this study implies a low prevalence of LF in the Ethiopian regions studied, suggesting that achieving Global Programme to Eliminate Lymphatic Filariasis (GPELF) targets through focused morbidity strategies is feasible. However, it is crucial to acknowledge that owing to the significant stigma associated with hydrocele [30], the reported numbers in this study may underestimate the actual prevalence. Given that HEWs are likely part of the same community as patients are [31], some individuals might choose not to disclose their condition to them. To address both the identified hydrocele cases and those potentially concealed, it is essential to establish inclusive pathways for referral and ensure access to safe hydrocele surgeries for condition correction.

This study has some limitations. It relied on the clinical identification of lymphedema and hydrocele, which could have led to misclassification in some cases, and confirmatory evaluations were performed. These evaluations were limited to a random sample, which may not fully capture the accuracy of all HEW-identified cases.

## Conclusion

This study successfully mapped the geographical distribution of LF morbidities across 24 LF-endemic districts in Ethiopia, identifying a substantial number of lymphedema and hydrocele cases, particularly in rural areas where healthcare access is limited. The findings highlight the importance of leveraging Ethiopia’s health extension program to identify affected individuals and ensure that they receive necessary care. The data collected can help inform targeted interventions and improve access to MMDP services in these regions, contributing to Ethiopia’s efforts to eliminate LF by 2027.

## Data Availability

The dataset supporting the conclusions of this article is included within the article and its additional files. Any additional material can be obtained upon reasonable request.

## Abbreviations

MoH: Ministry of health
GPELF: Global program to eliminate LF
HEW: Health extension worker
LF: Lymphatic filariasis
MMDP: Morbidity management and disability prevention
NaPAN: National Podoconiosis Action Network
NTD: Neglected tropical disease
MDA: Mass drug administration
PHCU: Primary health care unit
WHO: World Health Organization
